# Analysis of catch rates of LED lamps using on the falling-net fishing vessels in South China Sea

**DOI:** 10.1371/journal.pone.0301434

**Published:** 2024-04-04

**Authors:** Chunxi Wang, Qingxiang Chen, Zhengye Xiong, Zhiyu Chen, Rongchun Ye

**Affiliations:** 1 School of Electronic and Information Engineering, Guangdong Ocean University, Zhanjiang, Guangdong, China; 2 College of Ocean and Meteorology, Guangdong Ocean University, Zhanjiang, Guangdong, China; Universita degli Studi di Bari Aldo Moro, ITALY

## Abstract

Falling nets are a type of fishing gear that appeared and developed rapidly in the northern of South China Sea in the early 1990s. We have developed Light-emitting diode (LED) fishing lamps to replace metal halide (MH) lamps that reduce fuel consumption without reducing the catches. We conducted marine light-fishing experiments in the northern South China Sea during September 20 to 26, 2019 and August 29 to 31, 2021. The results in the first fishing experiment show that there is no significant change in catch of the falling-net fishing vessel when the white LED lamps (with a total power of 36 kW) were used instead of MH lamps (with a total power of 120 kW). Coleoidea catches of the falling-net fishing vessel increased significantly when white LED lamps (with a total power of 36 kW) and cyan LED lamps (with a total power of 6.0 kW) were used. The results in the second fishing experiment show that the total weight of catches of the cyan LED fishing lamps is more than that of the white LED fishing lamps, and the cyan LED light can attract *Penaeus merguiensis*, *Thryssa dussumieri* and *Sardinella zunasi* more effectively than the white LED light.

## 1. Introduction

For thousands of years, starting with bonfires on a beach or a boat, people have used artificial light to attract fish to improve catches [1], and this technique continues today as an important part of purse seining, lift netting, squid jigging, drop netting, and hook-and-line fishing [2]. Fishing with lights has been one of the most advanced and successful methods for catching squid, hairtail, anchovy, and other phototactic fishes for centuries [3–8].

Falling nets are a type of fishing gear that appeared and developed rapidly in the northern South China Sea in the early 1990s [9]. Falling-net fishing mainly aims to catch cephalopods and phototactic fishes. At present, falling-net fishing vessels have been widely distributed in the northern fishing ports of the South China Sea, research shows that the development of light fishing in the waters off the South China Sea is rapid, with the number of fishing boats in the spring flood season increasing from around 400 in 2012 to over 2000 in 2020 [10]. The fishing is mainly concentrated in coastal waters below 100 m. Strong artificial lights (usually metal halide (MH) lamps) are used on falling-net vessels to attract fish close to the fishing vessel. Most fishermen believe that stronger lights lead to larger catches and there has been a competition that has gradually increased the power of these lights.

Increasing fishing-light power means more fuel consumption. Fuel consumption by the world’s capture fisheries in 2000 was ~50 billion liters and this accounted for 1.2% of the global fuel consumption [11]. Owing to a rapid rise in fuel prices after 2004 and increasing concerns regarding greenhouse gas emissions [12], various studies have been conducted to reduce fuel consumption in capture fisheries [13]. Light-emitting diode (LED) lamp technology provides lower energy consumption, longer lifespan, higher efficiency, better chromatic performance, and reduced environmental impacts compared to traditional lighting technology [2]. The performance of LED lamps among commercial fisheries is known to vary across gear types and species. Using LED lamps can reduce fuel consumption, and catch variation exist as a result of differences in light characteristics, such as wavelength or intensity, or its application that could affect underwater irradiance or spatial distribution [3, 5, 6, 8, 14–16].

Several studies have demonstrated the economic and environmental benefits of using artificial light in other fisheries [3, 5, 15]. These studies have shown that a key challenge in adopting LED lamps is ensuring a return on investment and increased profit thereafter, LED fishing lamps are greener technological orientation and consume less energy than MH lamps [11, 13]. We have developed LED fishing lamps to replace MH lamps. In order to investigate the performance of LED fishing lamps replacing metal halide lamp and verify the best LED fishing lamp among the LED fishing developments, we conducted two marine fishing experiments. The experiments compared fishing performance between operations using traditional MH lamps to those using LED lamps on the falling-net vessel fishing in the northern South China Sea.

## 2. Materials and methods

### 2.1 Study area

All the fishing experiments are conducted on two falling-net fishing vessels. The fishing vessels are ~31 m long, ~5.6 m wide, and ~2.9 m deep, with a main engine power of 220.5 kW. There are three motors with powers of 180, 60, and 30 kW, respectively on each vessel. The fishing vessels were equipped with 120 MH fishing lamps (×1 kW) before 2018.

**Fig 1:**
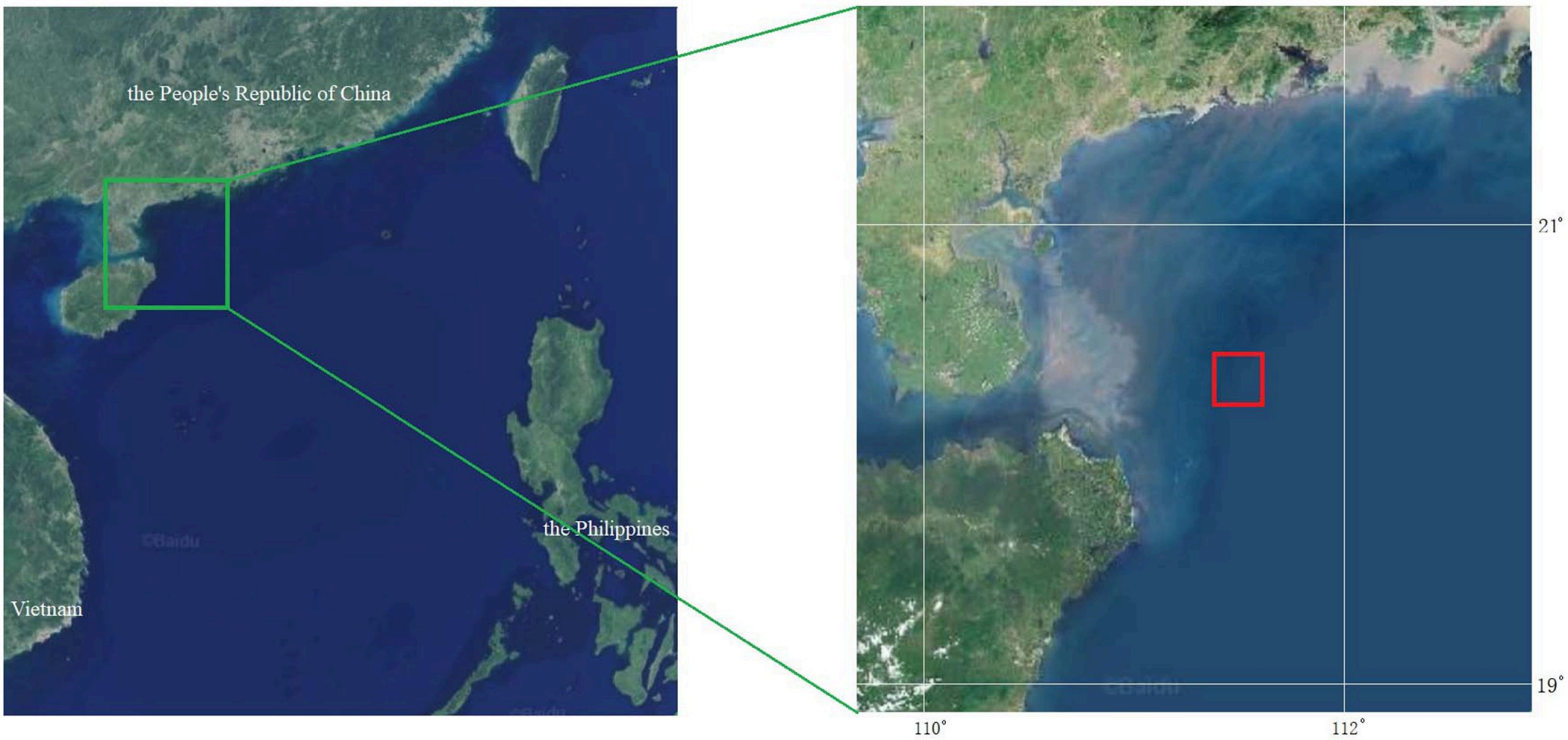
Maps showing the experimental area (within the red rectangular box, reprinted from https://map.baidu.com/@12425251.164467698,1829181.2786919929,6.05z/maptype%3DB_EARTH_MAP under a CC BY license, with permission from Baidu, Inc).

The first experiment was conducted from September 20 to 26, 2019, and the second experiment was conducted from August 29 to 31, 2021. The experimental area was in the northern South China Sea, Guangdong province, near 20° 25’N and 111°49’E (Fig 1). The water depth in this area is about 100~150 meters, and the water flow is gentle, making it suitable for falling-net fishing vessels.

### 2.2 LED light features

**Fig 2:**
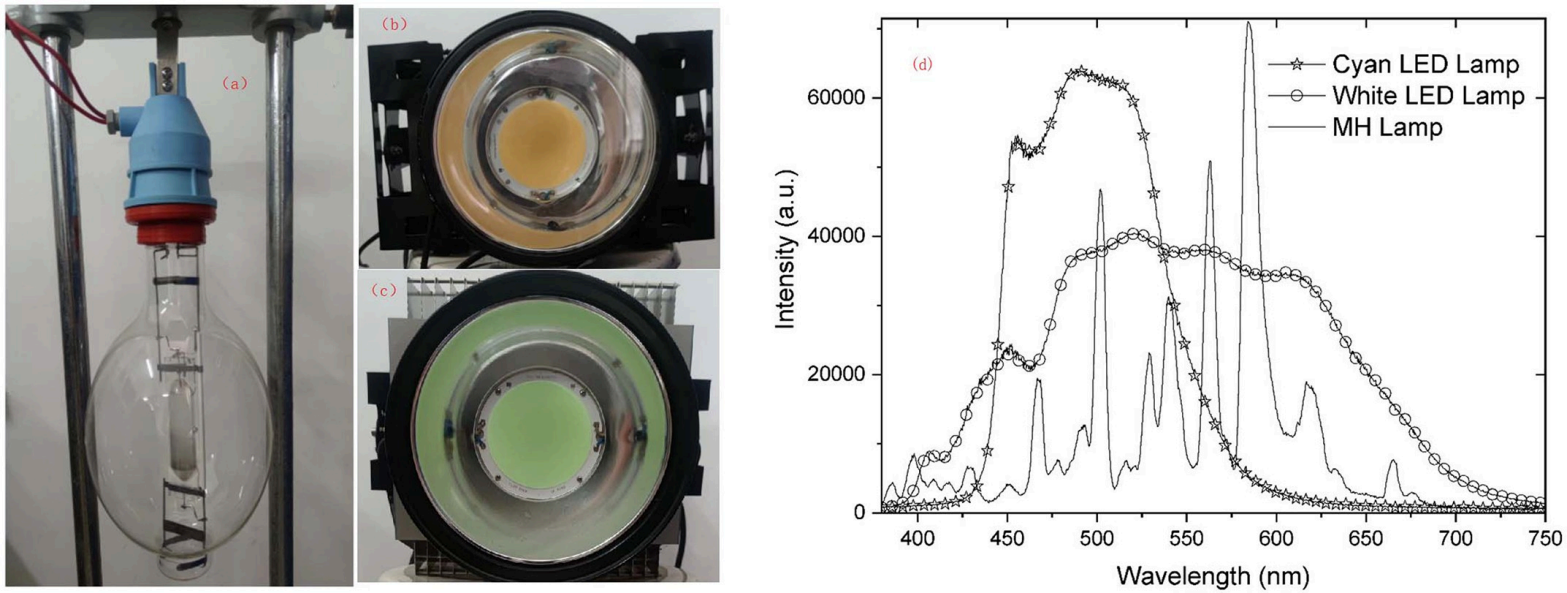
Spectra of LED fishing lamps and MH lamp. a. MH lamp, b. White LED fishing lamp, c. Cyan LED fishing lamp, d. Spectra of the fishing lamps.

Three types of fishing lamps were used in the experiments: metal halide (MH) lamps (Fig 2(a)) (manufactured by Giant Fish Lighting), white LED lamps (Fig 2(b), and cyan LED lamps (Fig 2(c)) (manufactured by Flip Chip of King, Zhongshan, China). The power of MH lamp is 1000W, and the power of LED lamp is 300W. We measured the distribution of spectral wavelengths released from the LED lamps using a spectrometer (Ocean Optics USB2000+). The spectra of the white LED and cyan LED fishing lamps (300 kW) are shown in Fig 2(d), and the spectra were measured with the fiber optic probe approximately 5 meters in front of the lamps. As can be seen that the white light LED fishing lamp covers almost the entire visible range, with significant luminescence in the range of 380–720 nm and strong luminescence in a wide range of 430–680 nm. The color temperature of white light is ~4800°C, which is very close to that of sunlight in the afternoon. The cyan light LED fish lamp has a significant luminescence in the range of 430–580 nm. The spectra of metal halide lamp measured under similar conditions are also shown in Fig 2d.

The brightness test experiment results show that the brightness of a single 1000W metal halide lamp is about 83404 lumens, with a lumen efficiency of about 83.4 Lm/W. The brightness of a single 300W cyan LED lamp is about 25663 lumens, with a lumen efficiency of about 85.5Lm/W. The brightness of a single 300W white LED lamp can reach 35266 lumens, with a lumen efficiency of about 117.6Lm/W.

Since the light emitted from metal halide lamps is scattered in all directions, more than half of the light cannot reach the sea surface, while the emission of LED lights can be controlled in a certain angle, and most light from LED fishing lights can shine towards the sea surface. Although the brightness of LED fishing lamps is much lower than that of metal halide lamps, the illumination of single cyan LED fishing lamps at 5 meters can reach over 327 Lx, the illumination of single white LED lamps at 5 meters can reach over 449 Lx, while the illumination of single metal halide lamp at 5 meters can only reach 266Lx. Therefore, for the effect of lighting, LED fishing lamp (*P* = 300 W) can replace metal halide lamp (*P* = 1000 W).

### 2.3 Experiment design and sampling procedures

**Fig 3:**
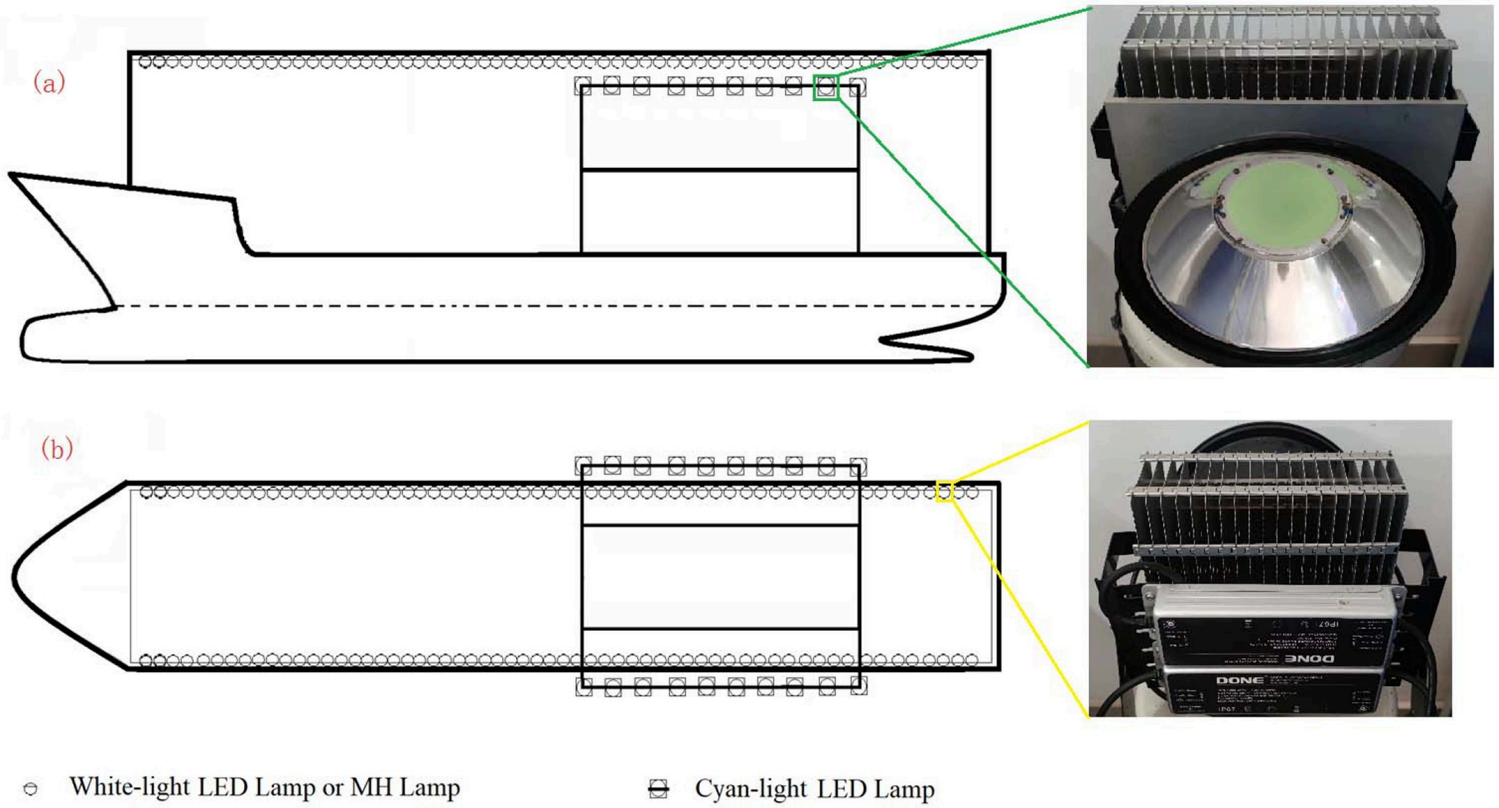
Fishing lamps fixed on the falling-net fishing vessel. a. an elevation view, b. a top view.

A total of 120 MH lamps (power *P* = 1 kW) were used on the nights of September 20 and 21, 2019, and the lamps were fixed vertically on two cross bars above the vessel, as shown in Fig 3. 120 white LED fishing lamps (*P* = 300 W) were used on the nights of September 23 and 24, 2019, and the LED lamps were fixed at almost the same position as the MH lamps. The direction of luminous COB (Chip On Board) of the LED lamps was downward, diverged at about 30 degrees from the horizontal direction, as shown in Fig 3b; in addition to the 120 white LED fishing lamps, 20 cyan LED fishing lamps (*P* = 300 W) were fixed on the vessel side on the nights of September 25 and 26, and the direction of luminous COB of the cyan LED lamps was downward diverging at about 45 degrees from the horizontal direction, as shown in Fig 3a. The fishing lights were turned on to gather fish at ~8 pm every night. Four net-fishing operations could be completed every night, which means that we had eight net catches under each lighting condition. After each net collection, the catches were classified and weighted, and species and weight of catches were recorded.

The second experiment was conducted from August 29 to 31, 2021, in order to compare the fishing effect of cyan light LED lamp and white light LED lamp. White LED lamps and cyan LED lamps are equipped on two ships respectively, and the fixed positions are almost the same (fixed on the two cross bars above each vessel). The total power of LED lights on each vessel is 36 Kw. All the LED lamps are manufactured by Flip Chip of King, Zhongshan, China. The experimental sites of the two vessels are about 3 kilometers apart. The fishing lights were turned on to gather fish at ~8 pm every night. Four net-fishing operations could be completed every night, which means that we had eight net catches under each lighting condition. After each net collection, the catches were classified and weighted, and species and weight of catches were recorded. The index of relative importance (IRI) was used to analyze the dominant species caught with different fishing lamps, and the IRI of the dominant species is not less than 1000 [17].

$$IRI=\left(n_{i}/N+w_{i}/W\right)\cdot F_{i}\cdot 10^{4}$$ (1)

Where, *n*_*i*_ and *w*_*i*_ are the mantissa and mass of the catch respectively, *N* and *W* are the total mantissa and total mass of all catches respectively, and *F*_*i*_ represents the percentage of occurrence frequency.

### 2.4 Statistical analysis

#### 2.4.1 Catch analysis of the first fishing experiment

Identification revealed that the number of obtained species under the three kinds of lighting were almost the same, being ~25 species. Among the numerous species, hairtail (*Trichiurus brevis*), bonito (*Auxis thazard*), mackerel (*Polydactylus sextarius*), coleoidea (*Loligo chinensis*), moonfish (*Mene maculata*), and *Carangidae* (*Decapterus maruadsi*, *Trachurus japonicus*, and *Selaroides leptolepis*) were more common in the first fishing experiment process.

The catch can be roughly divided into five categories: *Carangidae*, hairtail, coleoidea, *Scombrida*, and other fish in first fishing experiment. The main species of *Carangidae* were *Decapterus maruadsi*, *Trachurus japonicus*, and *Selaroides leptolepis*, and the catch of *Carangidae* also included *Caranx kalla*, *Caranx djeddaba*, *Ilisha melastoma*, *Chorinemus tolooparah*, and *Selar curmenophthalmus*. The main species of hairtail were *Trichiurus brevis* and *Trichiurus lepturus*. The main species of coleoidea were *Loligo chinensis* and *Sepiella maindroni*. The main species of *Scombrida* were *Auxis thazard* and *Rastrelliger kanagurta*; other species included *Polydactylus sextarius*, *Argyrosomus argentatus*, *Mene maculata*, and *Thamnaconus modestus*. The catches are listed in Table 1, the data of each net catch can be found in the document "Data of the First Experiment".

**Table 1:** Catches from the gathering methods using three light sources.

Catches	With MH lamps (*P* = 120 kW)		With white LED lamps (*P* = 36 kW)		With white and cyan LED lamps (*P* = 42 kW)	
	Average weight (kg)	S.D. (kg)	Average weight (kg)	S.D. (kg)	Average weight (kg)	S.D. (kg)
Carangidae	36.5	23.6	29.8	20.5	44.8	26.5
Trichiuridae	20.2	4.7	22.6	5.8	24.8	4.6
Coleoidea	19.6	4.3	19	3.5	24.8	3.2
Scombrida	17.8	7.9	21.6	9.4	20.2	9.6
Other species	13.5	3.8	14.8	4.0	16.2	4.2

According to the Shapiro-Wilk test [18], most of the catch data in the first experiment followed a normal distribution, but there were still some catching data inconsistent with a normal distribution. the data of coleoidea catches obtained by white light LED lamps and that of total catches obtained by MH lamps are inconsistent with normal distribution, and the p-values of the data are 0.044, less than 0.05; the data of total catches obtained by white LED lamps are inconsistent with normal distribution, and the p-value of the data is 0.026. The rank-sum test [19] is an appropriate method to discuss the significant differences between the catching data by different lamps. The Kruskal-Wallis test [20] was used to calculate the significance of inter-group difference of the catches under three lighting conditions. The results showed that there was no significant difference in the data of catches except for that of coleoidea. The Mann-Whitney U test [21] was used to determine the significant difference in the data of coleoidea catches between different lighting conditions. The results showed that there was no significant difference in coleoidea catches between under MH-lamp lighting and under white-LED-lamp lighting, but there was a significant difference in coleoidea catches between under white & cyan LED lamp lighting and under the other two lighting conditions.

The Mann-Whitney U test was used to calculate significance of coleoidea catches between under White&Cyan LED lamps lighting condition and under the other condition. The results show that the Rank Sum of the Data of white LED’s Catch is 42, while that of white & cyan LED is 94. The statistic of Mann-Whitney U test is 6, and the progressive significance (P) is 0.006; the Rank Sum of the Data of MH lamp’s Catch is 44, while that of white & cyan LED is 92, the statistic of Mann-Whitney U test is 8, and the progressive significance (P) is 0.012.

#### 2.4.2 Catch analysis of the second fishing experiment

After identification, there is little difference between the catch species of the white LED fishing lamps and that of the cyan LED fishing lamps. There are 37 catch species of the white LED fishing lamps and 36 catch species of cyan LED fishing lamps (the data can be found in the document "Species and Probability of Catches").

The data of each net catch can be found in the document "Data of the Second Experiment". Among the numerous catching species, hairtail (*Trichiurus brevis*), bonito (*Auxis thazard*), mackerel (*Polydactylus sextarius*), shrimp (*Penaeus chinensis*), moonfish (*Mene maculata*), and *Carangidae* (*Decapterus maruadsi*, *Trachurus japonicus*, and *Selaroides leptolepis*) were more common in the second fishing experiment process.

According to the SW test [18], the catch data in the second experiment basically conform to normal distributions. Both rank-sum test [19] and T-test [22] can be used to confirm significant differences in inter-group data. The dominant fishing catches are shown in Table 2, and the IRIs in the table are obtained by random sampling method. The main catches (accounting for more than 5% by weight) with white LED lamps are *Loligo chinensis* (33.86 wt%), *Caranx kalla* (20.75 wt%), *Sardinella aurita* (14.58 wt%), *Sardinella zunasi* (7.65 wt%), *Decapterus maruadsi* (8.31 wt%), while the main catches with cyan LED lamps are *Loligo chinensis* (18.57 wt%) and *Caranx kalla* (15.88 wt%), *Sardinella zunasi* (18.07 wt%), *Thryssa dussumieri* (5.03 wt%), *Penaeus merguiensis* (21.58 wt%). The Mann-Whitney U test [21] was used to determine the significant differences in catch data under difference lighting conditions. The results showed that there was no significant difference in *Caranx kalla* catches under white LED lamp lighting and under cyan LED lamp lighting, while there are significant differences in the data of *Loligo chinensis*, *Sardinella zunasi*, and Total catches, and the corresponding P-values are 0.046, 0.002, and 0.036, respectively.

**Table 2:** Common dominant species of 2 kinds of fishing lamps.

Fishing lamps	Species	IRI	Average weight (Kg)	S.D. of the weight (Kg)	Weight ratio
White LED fishing lamps	*Caranx kalla*	6151.2	15.2	5.2	20.75%
	*Loligo chinensis*	5862.0	24.7	7.7	33.86%
	*Sardinella aurita*	2375.4	10.6	3.8	14.58%
	*Sardinella zunasi*	1883.1	5.6	2.4	7.65%
	*Decapterus maruadsi*	1664.7	6.1	1.5	8.31%
	Other species	2063.6	10.8	10.4	14.85%
	Total catches	20000.0	73.0	16.5	100%
Cyan LED fishing lamps	*Caranx kalla*	3746.1	14.9	3.6	15.88%
	*Penaeus merguiensis*	3677.7	20.3	5.8	21.58%
	*Sardinella zunasi*	3519.5	17.0	6.5	18.07%
	*Loligo chinensis*	2527.2	17.4	4.9	18.57%
	*Thryssa dussumieri*	1009.0	4.7	1.8	5.03%
	Other species	5520.5	19.6	16.7	20.88%
	Total catches	20000.0	93.9	18.7	100%

The significance of variance between the catches of *Caranx kalla* with white LED lamps and with cyan LED lamps was calculated using the independent sample T-test method [22], the result show that the Sig. of variance is 0.331 (*p* = 0.331>0.05), and the Sig. of mean is 0.912 (*p* = 0.912 >0.05, *t* = 0.112), consistent with the result of the rank sum test, indicating that there is no significant difference in *Caranx kalla* catch between the two operating methods. *Sardine* displays a strong phototaxis [23] and the second fishing experiment results show that *Sardinella aurita* prefers white light, while *Sardinella zunasi* prefers cyan light. The significance of variance between *Sardinella zunasi* catches with white LED lamps and with cyan LED lamps was calculated using the independent sample T-test method, the result show that the Sig. of variance is 0.047 (*p* = 0.047≈0.05), and the Sig. of mean is 0.001 (*p* = 0.001<0.05, *t* = 4.662); the significance of variance between the total catches with white LED lamps and with cyan LED lamps was calculated using the independent sample T-test method, the result show that the Sig. of variance is 0.764 (*p* = 0.764>0.05), and the Sig. of mean is 0.033 (*p* = 0.033<0.05, *t* = 2.369). These results consist with the results of the rank sum tests, indicating that there are significant differences in *Sardinella zunasi* and Total catches between the two operating methods. Combine with the data in Table 2, it can be concluded that Total catches and Sardinella zunasi can be increased under cyan LED lamps lighting.

The significance of variance between *Loligo chinensis* catches with white LED lamps and with cyan LED lamps was calculated using the independent sample T-test method, the result show that the Sig. of variance is 0.286 (*p* = 0.286>0.05), and the Sig. of mean is 0.04 (*p* = 0.033<0.05, *t* = 2.369). The result consists with that of the rank sum tests, indicating that there is a significant difference in *Loligo chinensis* catches between the two operating methods. Combine with the data in Table 2, it can be concluded that *Loligo chinensis* catches will reduce slightly under cyan LED lamps lighting.

## 3. Discussion

### 3.1 The feasibility of replacing MH lamps with white LED fishing lamps

The aim of this study was to find a high-efficiency method to fish in the ocean with LED fishing lamps on falling-net fishing vessels. The results show that catches are similar using white LED fishing lamps compared with using MH lamps, but the power of white LED fishing lamps is only one third of that of MH lamps. This result is consistent with the report of Li et al. [24]. It can be seen from Fig 2 that the light emitted by white LEDs is similar to the light emitted by MH lamps, except that the glowing peak of the MH lamps is sharper than that of the LED lamps. The spectrum of the white LED lamp basically encompasses the spectrum of the MH lamp in the visible-light range.

The lumen efficiency of the white LED lamps used was 117.6 Lm/W, and that of the MH lamps was 83.4 Lm/W. MH lamps are usually suspended vertically, and their luminescence is rotationally symmetric. Therefore, only ~1/6 of their light can project to the sea surface. Although the power of the white LED fishing lamps (300 W) was less than that of the MH lamp (1 kW), the white LED fishing lamp can effectively project >60% of its light to the sea surface because of its better light-emitting direction. Therefore, the illuminating effect of white LED fishing lamps can be almost the same as that of MH lamps. Based on a reasonable design, 120 white LED lights were installed on the falling-net vessel to replace the original 120 MH lights.

It can be seen from the result of the first experiment that not all the catch data can follow normal distributions, and both Kruskal-Wallis test and Mann- Whitney U test confirm that there is no significant difference in the catches between under MH lamps lighting and under white LED lamps lighting. So it is feasible to replace MH lamp of 1000 W with white LED fishing lamp of 300 W.

### 3.2 The fish attracting effect of light is related to the optical characteristics of water

In our experiments in coastal waters, we used white-light LED lamps and blue-light LED lamps separately to attract *Trachurus japonicus* and *Decapterus maruadsi* [25]. The results demonstrate that white-light LED lamps have better attractive effects on *Carangidae* than blue-light LED lamps at equal power ratings. This result is basically consistent with the results obtained by Lin et al. [26], although some experimental results have confirmed that blue light has a better attraction effect on fish [27].

**Fig 4:**
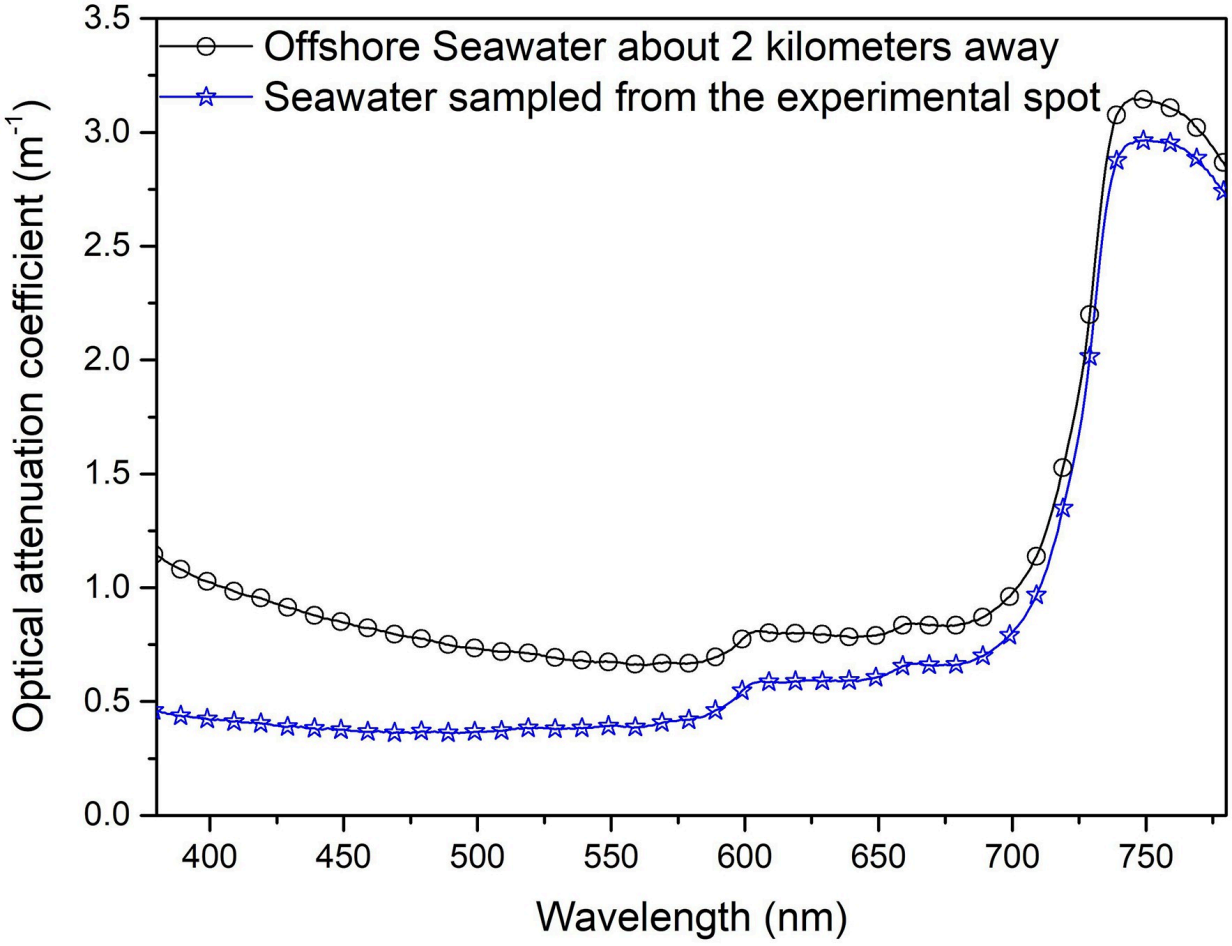
Optical attenuation coefficient of coastal seawater and experimental spot seawater.

We measured the light attenuation coefficients of seawater sampled 2 km from the coast and at the experimental spot, respectively, using a Shimazdu UV-2600 spectrophotometer. As shown in Fig 4, coastal seawater exhibits a light attenuation coefficient (< 1.0 m^−1^) at a waveband of 410–700 nm, which is the principal light waveband of white-light LED lamps. The light attenuation coefficients average 0.7843 m^−1^ in this light waveband. Blue-light LED lamps, which have a wavelength range of 410–450 nm, centered at 430 nm, have few advantages in terms of penetrating depth in coastal waters in comparison with white-light LED lamps, which have a wavelength range of 410–700 nm and an average optical attenuation coefficient of 0.9116 m^−1^.

The illuminance variation as a function of depth as the light irradiates the sea surface can be estimated by using [28]

$$E\left(h\right)=E_{0}\text{exp}\left(-\alpha h\right).$$ (2)

where *E*(*h*) is the illuminance at depth *h*, *E*_0_ is the illuminance at the sea surface, and *α* is the optical attenuation coefficient.

The average optical attenuation coefficient of the coastal seawater (sampled 2 km from the coast) at a waveband of 410–700 nm is less (~0.7843m^-1^) than that at a waveband of 410–450 nm (~0.9116), that means the white-light LED lamp has a greater illuminance effect than the blue-light LED lamp. This is one of the reasons for some vessels using white-light LED lamps exhibited a significantly greater fish catches than that using blue LED lamps in coastal areas.

When the experiment is carried out in clean seawater (sampled from the experimental spot), the results are figured in Fig 4, for the seawater sampled from the experimental spot the average optical attenuation coefficient at a waveband of 410–700 nm is more (~0.4757 m^-1^) than that at a waveband of 410–450 nm (~0.3918 m^-1^), that means the blue-light LED lamp has a greater illuminance effect than the white-light LED lamp. The luminance efficiency of blue LED is generally slightly less than that of white LED, Therefore, the fish attracting effect of blue LED fishing lamp in clean seawater is not significantly different from that of white LED fishing lamp [29]. It can be seen that the fish attracting effect of light is not only related to the species of fish, but also related to the optical characteristics of water.

### 3.3. The cyan LED fishing lamp has better attracting effect to most fish than the white LED fishing lamp

**Fig 5:**
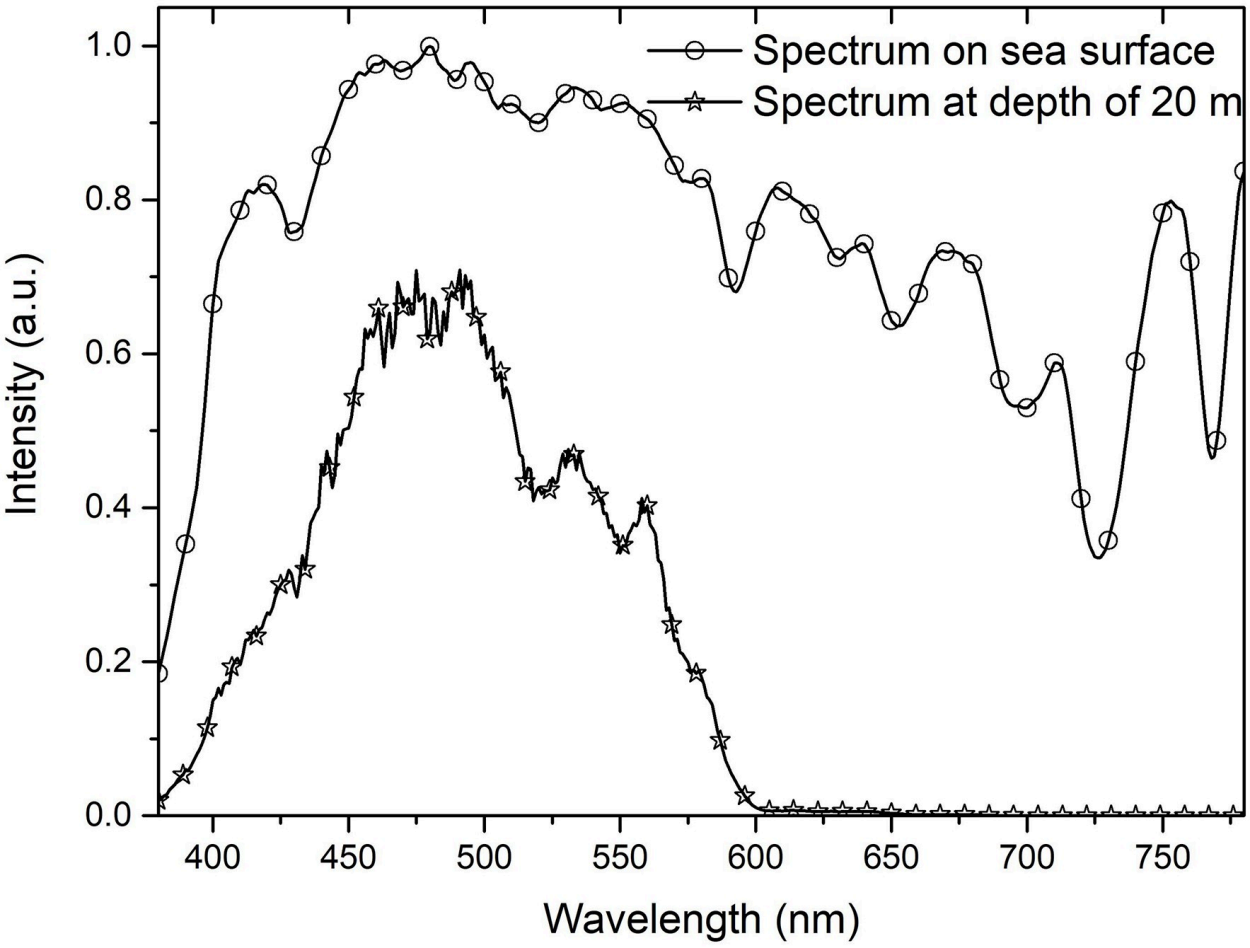
Spectrum of sunlight at the sea surface and that of light at a depth of 20 m.

The seawater at the experimental spot had an average beam attenuation coefficient of ~0.4757 m^−1^ for the white LED lamp waveband (410–700 nm) (Fig 4). The wavelength range for the cyan LED lamp used in the experiment was 430–600 nm, and the average beam attenuation coefficient at the waveband (430–600 nm) is 0.3915 m^−1^. The attenuation coefficient of cyan light in seawater is less than that of white light, and the light from the cyan LED fishing lamp can travel farther than that from the white LED fishing lamp in seawater. Fig 5 depicts the light spectra at the sea surface and at a depth of 20 m at ~11:00 am on a cloudy day as measured with an Emfine SFIM-300 spectrometer with waterproof treatment. The illuminance was ~3550 lx at the sea surface and ~1 lx at 20 m below the sea surface. As Fig 5 demonstrates, the peak wavelength of light shining on fish at a depth of 20 m was ~490 nm while the effective wavelength range was 400–590 nm. In other words, most fish species living at the depth of ~ 20 m in seawater are used to light in the wavelength range (400–590 nm). This may be one of the reasons why blue and green fish aggregation devices are more attractive to fish [26].

The addition of cyan LED lamps increased the total catch but not significantly in the first fishing experiment. In the first fishing experiment, the power of the cyan LED lamp increased by only 16.7%, but the weight of fishing catches increased by 21.3%. The Kruskal-Wallis test and Mann-Whitney U test confirm that the addition of cyan LED lamps increased coleoidea catch significantly. Due to the fact that the coleoidea catch data of MH lamps and white&Cyan LED lamps follow normal distributions, T-test can be used to confirm significant differences between two group data. The significance of variance between coleoidea catches with MH lamps and with white&cyan LED lamps calculated using the independent sample T-test method was 0.419>0.05, and the mean significance is 0.016<0.05. The average weight of coleoidea catch with MH lamps is 19.6±4.3, while that with white&cyan LED lamps is 24.8±3.2.

In the second experiment, the catch species of the cyan LED fishing lamps are similar to that of the white LED fishing lamps, but there are great differences in the total weight of catches and dominant catch species. The cyan LED fishing lamps has better aggregation effect to *Penaeus merguiensis*, *Thryssa dussumieri* and *Sardinella zunasi*, and the total weight of the cyan LED fishing lamps is significantly higher than that of the white LED fishing lamps The results are consistent with the experimental results reported in some other literatures [27, 30]. The average weight of total catch with white LED lamps is 73.0±16.5, while that with cyan LED lamps is 93.9±18.7.

### 3.4 Appropriate illumination is important in the falling-net fishing method

Replacing metal halide or incandescent lamps with LED lamps for fishing can save fuel and is better for the environment [31]. LED lights can improve the catchability of snow crab traps [32]. In the first experiment, adding 12 cyan LED fishing lamps significantly increased the catches of coleoidea; while in the second experiment, replacing 120 white LED fishing lamps with 120 cyan LED lamps significantly reduced the catches of coleoidea. Using 120 white LED fishing lamps, the average weight of *Loligo chinensis* per net is ~24.7 ±7.7 kg, while the average weight per net is ~17.4 ±4.9 kg using 120 cyan LED lamps. The significance of variance calculated using the independent sample T-test method is 0.286>0.05, indicating that there was no significant difference in variance between the two groups of data. The significance of the mean was 0.04<0.05, indicating that there is a significant difference in the mean of catches between the two operating methods. The visual nerves of coleoidea are sensitive to blue-green light [33]. In the first experiment, an appropriate cyan light increase into white light can significantly increase the catch of coleoidea, which is similar to the results of many other fish species [34, 35]. In the second experiment, replacing all white LED lamps with cyan LED lamps results in the cyan light in the water around the vessel was too strong, and *Loligo chinensis* may gather in a slightly dimmer range which is slightly farther from the fishing vessel. The effective- capture range of the falling-net fishing vessel is relatively smaller compared to the purse seine, and during the sinking process of the net, some *Loligo chinensis* near the edge of the effective-capture range of the fishing boat can easily escape. This may be the reason why in the second experiment, the total catches of *Loligo chinensis* with 120 light lamps decreased significantly. Excessive blue-green light may have a negative effect on increasing fishing catch, which is consistent with experimental results reported [1, 36].

## 4. Conclusions

Light-emitting diode (LED) fishing lamps is developed to replace metal halide (MH) lamps that reduce fuel consumption without reducing the catches. Marine light-fishing experiments were conducted in the northern South China Sea during September 20 to 26, 2019 and August 29 to 31, 2021.

The results in the first fishing experiment show that there is no significant change in catch of the falling-net fishing vessel when the white LED lamps (with a total power of 36 kW) were used instead of MH lamps (with a total power of 120 kW). There was no significant difference in total catch for the addition of cyan LED lamps (6.0 kW), but the catch of coleoidea increased from 19.0 ± 3.5 Kg per net to 24.8 ± 3.2 Kg. Coleoidea catches of the falling-net fishing vessel increased significantly when white LED lamps (with a total power of 36 kW) and cyan LED lamps (with a total power of 6.0 kW) were used.

The results in the second fishing experiment show that the total weight of total catches of the cyan LED fishing lamps is more than that of the white LED fishing lamps, and the cyan LED light can attract *Penaeus merguiensis*, *Thryssa dussumieri* and *Sardinella zunasi* more effectively than the white LED light.

The attenuation coefficient of cyan light in seawater is less than that of white light, and the light from the cyan LED fishing lamp can travel farther than that from the white LED fishing lamp in seawater. This may be one of the reasons why the cyan-light can be more attractive to most fish.

At present, cyan light LED fishing lamps are used on some falling-net fishing vessels (S1 Fig) in South China Sea, and help fishermen to save energy and increase their income (S2 & S3 Figs).

## Supporting information

S1 Fig Cyan light LED fishing lamps mounted on a falling-net fishing vessel. (TIF)

S2 Fig Pre harvest. A fisherman stood under the cyan fishing lamps, waiting to collect the falling-net.
(TIF)

S3 Fig The harvest. The weight of catches reached around 100 kilograms.
(TIF)
