# Supplementary figures and images for: Analysis of catch rates of LED lamps using on the falling-net fishing vessels in South China Sea
Source: PLoS One. 2024 Apr 4;19(4):e0301434. doi: 10.1371/journal.pone.0301434 (PMC10994366; doi:10.1371/journal.pone.0301434)

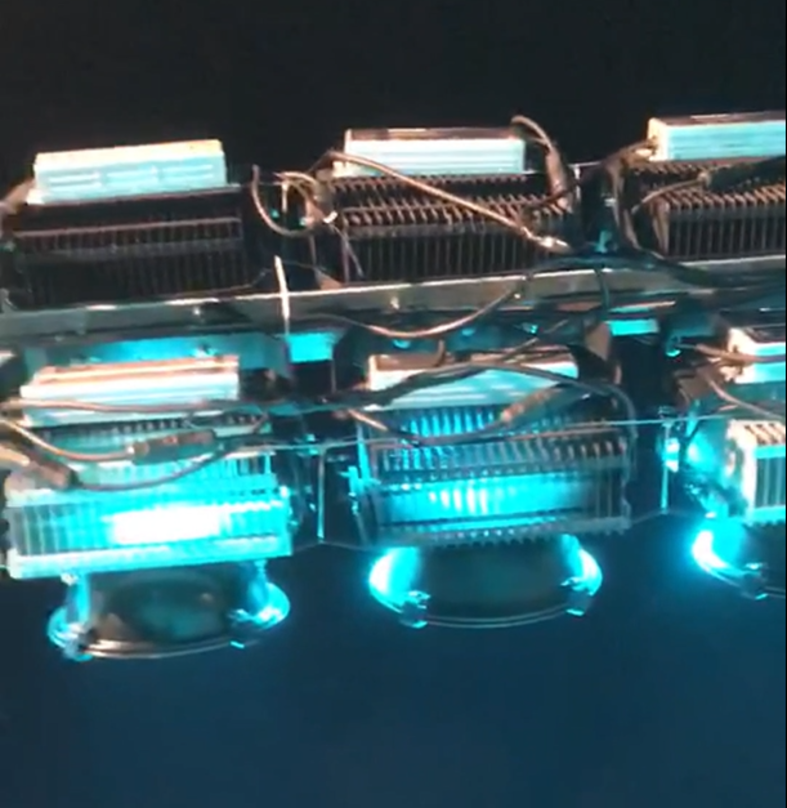

Supplement: S1 Fig — (TIF) [file pone.0301434.s001.tif]

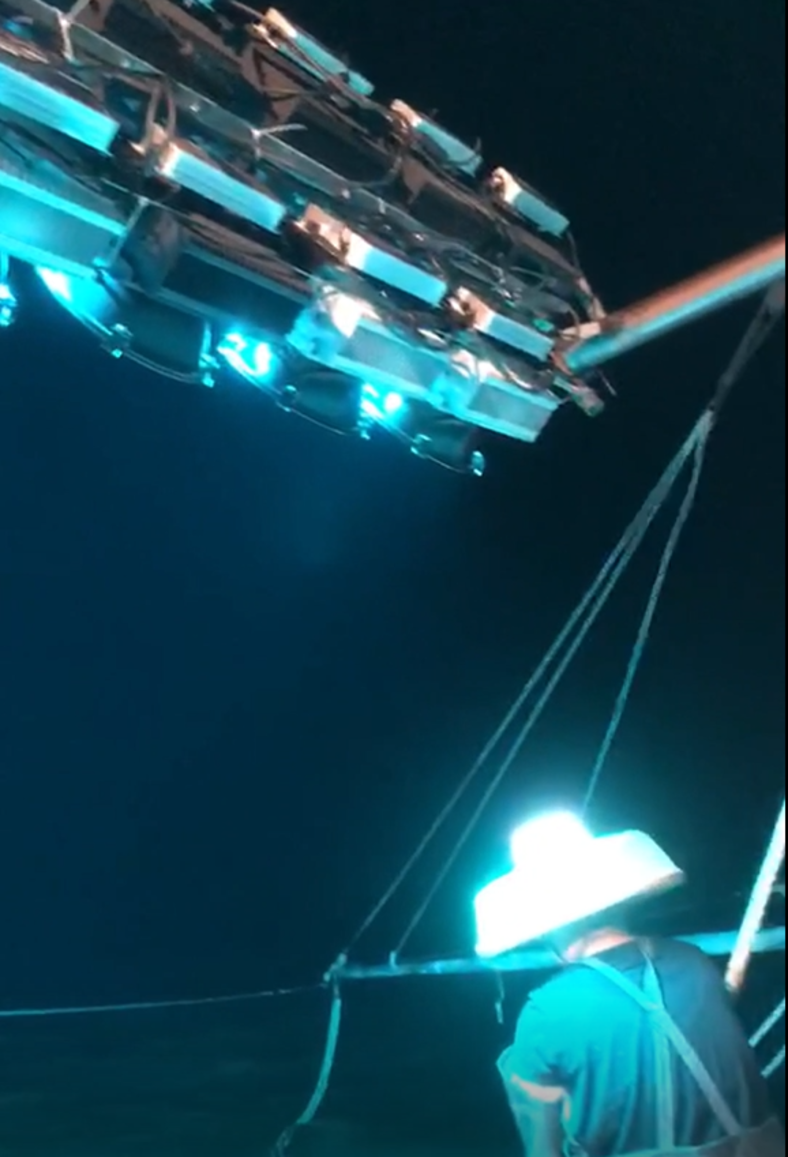

Supplement: S2 Fig — A fisherman stood under the cyan fishing lamps, waiting to collect the falling-net. (TIF) [file pone.0301434.s002.tif]

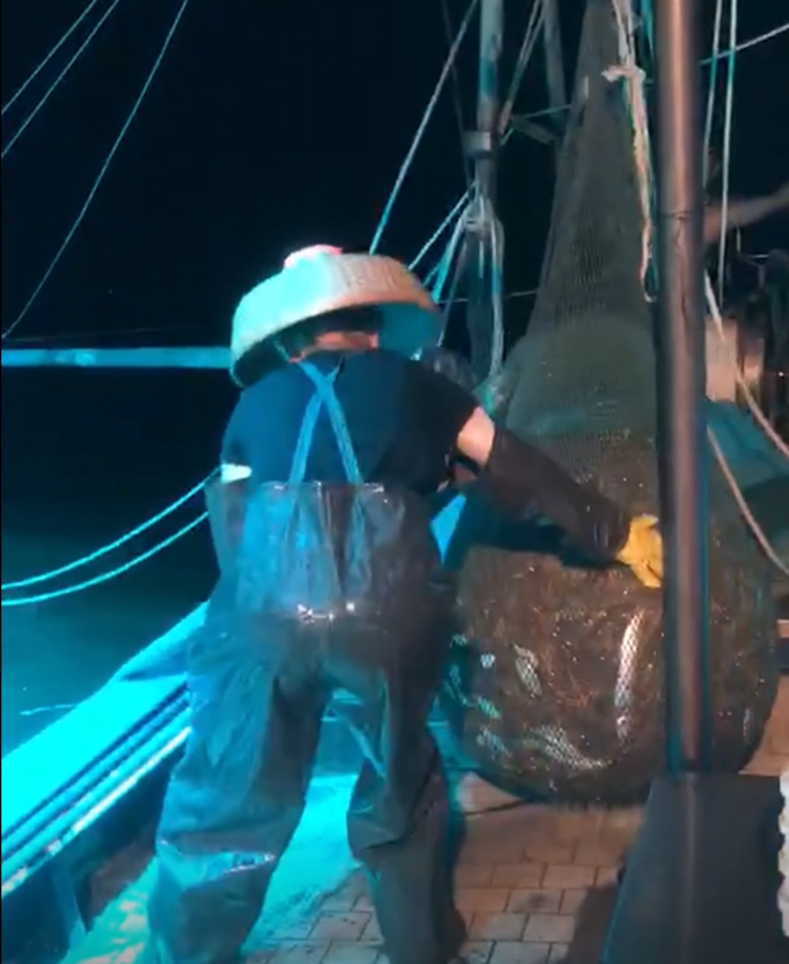

Supplement: S3 Fig — The weight of catches reached around 100 kilograms. (TIF) [file pone.0301434.s003.tif]
